# Supplementary material for: Evaluation of Microglia/Macrophage Cells from Rat Striatum and Prefrontal Cortex Reveals Differential Expression of Inflammatory-Related mRNA after Methamphetamine
Source: Brain Sci. 2019 Nov 25;9(12):340. doi: 10.3390/brainsci9120340 (PMC6955783; doi:10.3390/brainsci9120340)
Supplement: Supplementary file 1 [file brainsci-09-00340-s001.pdf]

**Supplementary Materials:** The following are available online at http://www.mdpi.com/2076-3425/9/12/340/s1, Table S1: Gene expression data for Figure 3, Table S2: Gene expression data for Figure 4, Table S3: Gene expression data for Figure 5, Table S4: Gene expression data for Figure 6, Table S5: Gene expression data for GABA transporters in microglia from the STR at 2 hr and 3 days after METH, Table S6: Gene expression data for *Csf1, Csfr1, Il34* in microglia from the STR at 2 hr and 3 days after METH.

Table S1: Gene expression data for Figure 3. PG and LT biosynthetic enzymes and LT receptors in 2 hr post METH STR and PFC microglia. ND = Not Detected. NS = Not Significant.

| Symbol | Gene Name | 2 hr STR average log2cpm<br><br>Saline (n=6), METH (n=8) | 2 hr STR<br><br>Fold Change, FDR | 2 hr PFC average log2cpm S<br><br>Saline (n=4), METH (n=5) | 2 hr PFC<br><br>Fold Change, FDR |
|---|---|---|---|---|---|
| Plaa | phospholipase A2, activating protein | 5.34, 5.95 | 1.55, 3E-04 | 5.34, 5.64 | 1.25, 0.08 (NS) |
| Ptgs1 | prostaglandin-endoperoxide synthase 1 (COX1) | 10.36, 9.70 | -1.55, 3E-05 | 10.15, 9.40 | -1.65, 7.0E-07 |
| Ptgs2 | prostaglandin-endoperoxide synthase 2 (COX2) | -4.73, -0.62 | 47.3, 2E-03 | ND | ND |
| Alox5 | arachidonate 5-lipoxygenase | 8.24, 8.11 | -1.06, 0.79 (NS) | 8.40, 8.98 | 1.51, 0.01 |
| Alox5ap | arachidonate 5-lipoxygenase activating protein | 7.67, 8.15 | 1.43, 5.5E-04 | 7.76, 8.49 | 1.68, 3.6E-06 |
| Lta4h | leukotriene A4 hydrolase | 7.16, 7.58 | 1.36, 4E-04 | 7.12, 7.65 | 1.46, 9E-06 |
| Ptgr1 | prostaglandin reductase 1 | 2.02, 0.91 | -1.98, 8.8E-04 | 2.40, 0.89 | -2.74, 7.0E-06 |
| Ltb4r | Leukotriene B4 Receptor | ND | ND | ND | ND |
| Ltc4s | leukotriene C4 synthase | 8.11, 6.91 | -2.1, 1E-05 | 8.11, 7.35 | -1.61, 9.0E-04 |
| Ggt1 | gamma-glutamyltransferase 1 | ND | ND | ND | ND |

| | | | | | |
|---|---|---|---|---|---|
| Ggt7 | gamma-glutamyltransferase 7 | 4.15, 3.65 | -1.38, 0.045 | 4.20, 3.60 | -1.55, 0.04 |
| Dpep2 | dipeptidase 2 | 3.59, 2.94 | -1.6, 6E-03 | 3.81, 3.05 | -1.72, 3.0E-04 |
| Cysltr1 | cysteinyl leukotriene receptor 1 | 4.48, 3.65 | -1.7, 9E-04 | 4.45, 4.06 | -1.3, 0.34 (NS) |
| Cysltr2 | cysteinyl leukotriene receptor 2 | ND | ND | ND | ND |
| P2ry12 | purinergic receptor P2Y12 | 10.88, 9.77 | -2.0, 5E-05 | 10.93, 10.23 | -1.6, 5.0E-05 |

Table S2: Gene expression data for Figure 4. Biosynthetic enzymes and receptors for PG's, TxA2, and HETE's in 2 hr post METH STR and PFC microglia. ND = Not Detected. NS= Not Significant.

| Symbol | Gene Name | 2 hr STR average log2cpm<br><br>Saline (n=6), METH (n=8) | 2 hr STR<br><br>Fold Change, FDR | 2 hr PFC average log2cpm<br><br>Saline (n=4), METH (n=5) | 2 hr PFC<br><br>Fold Change, FDR |
|---|---|---|---|---|---|
| Plaa | phospholipase A2, activating protein | 5.34, 5.95 | 1.55, 3E-04 | 5.34, 5.64 | 1.25, 0.08 (NS) |
| Ptgs1 | prostaglandin-endoperoxide synthase 1 (COX1) | 10.36, 9.70 | -1.55, 3E-05 | 10.15, 9.40 | -1.65, 7E-07 |
| Ptgs2 | prostaglandin-endoperoxide synthase 2 (COX2) | -4.73, -0.62 | 47.3, 2E-03 | ND | ND |
| Ptges2 | prostaglandin E synthase 2 | 4.62, 5.41 | 1.76, 8E-06 | 1.24, 0.14 (NS) | 4.63, 4.92 |
| Ptges3 | prostaglandin E synthase 3 | 6.70, 8.04 | 2.60, 3E-11 | 6.92, 8.18 | 2.43, 1E-07 |
| Ptger1 | prostaglandin E receptor 1 | 3.21, 3.86 | 1.61, 0.07 (NS) | 3.32, 3.91 | 1.36, 0.22 (NS) |
| Ptger2 | prostaglandin E receptor 2 | 0.32, 2.56 | 5.0, 2E-05 | 0.56, 1.82 | 2.50, 0.01 |
| Ptger3 | prostaglandin E receptor 3 | ND | ND | ND | ND |
| Ptger4 | prostaglandin E receptor 4 | 2.88, 5.19 | 6.5, 3E-05 | 3.16, 4.45 | 2.64, 6E-03 |

| | | | | | |
|---|---|---|---|---|---|
| Alox15 | arachidonate 15-Lipoxygenase | ND | ND | ND | ND |
| Alox12 | arachidonate 12-lipoxygenase, 12S type | 6.60, 6.13 | -1.36, 0.11 (NS) | 6.92, 5.29 | -2.95, 3.3E-07 |
| Alox5 | arachidonate 5-lipoxygenase | 8.24, 8.11 | -1.06, 0.79 (NS) | 8.40, 8.98 | 1.51, 0.01 |
| Alox5ap | arachidonate 5-lipoxygenase activating protein | 7.67, 8.15 | 1.43, 5.5E-04 | 7.76, 8.49 | 1.68, 3.6E-06 |
| Ppard | peroxisome proliferator-activated receptor delta | 3.63, 4.23 | 1.54, 0.04 | 3.60, 4.26 | 1.55, 0.01 |
| Pparg | peroxisome proliferator activated receptor gamma | ND | ND | ND | ND |
| Angptl4 | angiopoietin-like 4 | 0.94, 2.42 | 4.20, 1.2E-04 | 0.45, 2.39 | 3.78, 1.4E-07 |
| Gpr31 | G protein-coupled receptor 31 | 5.76, 3.30 | -4.25, 1.6E-05 | 5..61, 4.46 | -2.22, 4.3E-08 |
| Oxer1 | Oxoeicosanoid Receptor 1 | ND | ND | ND | ND |
| Tbxas1 | thromboxane A synthase 1 | 8.38, 8.17 | -1.16, 0.11 (NS) | 8.26, 7.92 | -1.28, 0.09 NS) |
| Tbxa2r | thromboxane A2 receptor | -1.11, -1.31 | 1.42, 0.53 (NS) | -1.07, -2.15 | -2.19, 0.21 (NS) |
| Ptgds | prostaglandin D2 synthase | ND | ND | ND | ND |
| Ptgdr | prostaglandin D2 Receptor | ND | ND | ND | ND |
| Prx12b | peroxiredoxin like 2B (prostamide/prostaglandin F synthase) | ND | ND | ND | ND |
| Ptgfr | Prostaglandin F Receptor | ND | ND | ND | ND |
| Ptgis | Prostaglandin I2 Synthase | ND | ND | ND | ND |
| Ptgir | prostaglandin I2 receptor | -2.27, -0.59 | 2.82, 0.15 (NS) | -1.48, -1.04 | 1.83, 0.43 (NS) |

Table S3: Gene expression data for Figure 5. Significantly changed RNAseq genes associated with NRF2-mediated oxidative stress response via IPA®.

| Gene Symbol - rat (Entrez Gene) | Entrez Gene Name | Expr Fold Change | Expr False Discovery Rate (q-value) |
|---|---|---|---|
| *Bach1* | BTB domain and CNC homolog 1 | 2.01 | 5.62E-03 |
| *Cct7* | chaperonin containing TCP1 subunit 7 | 2.95 | 1.54E-07 |
| *Dnaja1* | DnaJ heat shock protein family (Hsp40) member A1 | 27.39 | 2.09E-07 |
| *Dnaja4* | DnaJ heat shock protein family (Hsp40) member A4 | 37.00 | 3.84E-10 |
| *Dnajb1* | DnaJ heat shock protein family (Hsp40) member B1 | 37.32 | 2.82E-05 |
| *Dnajb4* | DnaJ heat shock protein family (Hsp40) member B4 | 6.25 | 2.76E-03 |
| *Dnajb7* | DnaJ heat shock protein family (Hsp40) member B7 | 4.93 | 5.19E-04 |
| *Dnajb11* | DnaJ heat shock protein family (Hsp40) member B11 | 2.59 | 1.47E-08 |
| *Dnajc3* | DnaJ heat shock protein family (Hsp40) member C3 | 2.78 | 4.17E-08 |
| *Enc1* | ectodermal-neural cortex 1 | 2.22 | 8.20E-04 |
| *Ephx1* | epoxide hydrolase 1 | -3.52 | 1.27E-03 |
| *Fosl1* | FOS like 1, AP-1 transcription factor subunit (Fra1) | 11.11 | 2.54E-03 |
| *Fth1* | ferritin heavy chain 1 | 2.42 | 2.44E-06 |
| *Ftl1* | ferritin light chain | 2.09 | 3.18E-05 |

| | | | |
|---|---|---|---|
| *Gclc* | glutamate-cysteine ligase catalytic subunit | 9.13 | 2.01E-08 |
| *LOC501110 (GSTA2)* | glutathione S-transferase alpha 2 | -3.50 | 6.20E-05 |
| *Hmox1* | heme oxygenase 1 | 74.84 | 1.53E-08 |
| *Hspb8* | heat shock protein family B (small) member 8 (Hsp22) | 4.86 | 1.72E-02 |
| *Jun* | Jun proto-oncogene, AP-1 transcription factor subunit | 3.28 | 2.61E-02 |
| *Maff* | MAF bZIP transcription factor F | 4.95 | 1.46E-02 |
| *Mafg* | MAF bZIP transcription factor G | 2.08 | 5.09E-06 |
| *Mafk* | MAF bZIP transcription factor K | 4.00 | 2.35E-04 |
| *Map2k6* | mitogen-activated protein kinase kinase 6 | -2.67 | 1.66E-05 |
| *Mgst3* | microsomal glutathione S-transferase 3 | -2.13 | 5.60E-05 |
| *Nfe2l2 (Nrf2)* | nuclear factor, erythroid 2 like 2 (Nrf2) | 2.97 | 1.33E-06 |
| *Nqo1* | NAD(P)H quinone dehydrogenase 1 | 11.63 | 6.32E-05 |
| *Pik3c2b* | phosphatidylinositol-4-phosphate 3-kinase catalytic subunit type 2 beta | 5.09 | 2.34E-04 |
| *Pmf1* | polyamine modulated factor 1 | -2.97 | 7.04E-08 |
| *Sod1* | superoxide dismutase 1 | 3.98 | 2.16E-11 |
| *Stip1* | stress induced phosphoprotein 1 | 16.07 | 3.34E-12 |
| *Txnrd1* | thioredoxin reductase 1 | 3.39 | 5.67E-05 |
| Additional gene expression data related to NRF2-mediated oxidative stress response via IPA® | | | |

| | | | |
|---|---|---|---|
| *Hsp90aa1* | heat shock protein 90, alpha (cytosolic), class A member 1 | 30.58 | 2.75E-09 |
| *Hsp90ab1* | heat shock protein 90 alpha family class B member 1 | 12.78 | 2.76E-11 |

Table S4: Gene expression data for Figure 6. Hypothesized pathway and consequences of glutamine uptake in 2 hr post METH STR and PFC microglia. ND = Not Detected. NS = Not significant.

| Symbol | Gene Name | 2 hr STR average log2cpm<br><br>Saline (n=6), METH (n=8) | 2 hr STR<br><br>Fold Change, FDR | 2 hr PFC average log2cpm<br><br>Saline (n=4), METH (n=5) | 2 hr PFC<br><br>Fold Change, FDR |
|---|---|---|---|---|---|
| *Slc7a6* | solute carrier family 7 member 6 | 6.67, 7.00 | 1.30, 0.02 | 6.69, 6.51 | -1.09, 0.59 (NS) |
| *Slc38a7* | solute carrier family 38, member 7 | 5.34, 6.05 | 1.70, 2.8E-04 | 5.39, 5.83 | 1.38, 0.02 |
| *Slc1a5* | solute carrier family 1 member 5 | 3.71, 5.50 | 3.52, 1.6E-03 | 3.69, 3.83 | 1.47, 0.42 (NS) |
| *Slc38a1* | solute carrier family 38, member 1 | -2.78, -2.73 | -1.20, 0.68 (NS) | -1.57, -2.03 | -1.36, 0.71 (NS) |
| *Slc1a3* | solute carrier family 1 member 3 (EAAT1, GLAST1) | 10.08, 9.33 | -1.61, 6.3E-06 | 10.09, 8.90 | -2.29, 8.3E-10 |
| *Slc1a2* | solute carrier family 1 member 2 (EAAT2, GLT1) | 0.33, 0.40 | 1.27, 0.61 (NS) | 0.08, 0.33 | 1.03, 0.97 (NS) |
| *Slc7a11* | solute carrier family 7 member 11 | -0.59, 1.11 | 4.22, 0.01 | -0.75, -0.37 | 1.39, 0.63 (NS) |

| | | | | | |
|---|---|---|---|---|---|
| *Gls* | glutaminase | 5.91, 5.92 | 1.02, 0.92 (NS) | 5.99, 5.80 | -1.17, 0.17 (NS) |
| *Gls2* | glutaminase 2 | 0.83, 1.50 | 1.58, 0.08 (NS) | 0.88, 1.49 | 1.49, 0.16 (NS) |
| *Glul* | glutamate-ammonia ligase | 10.26, 10.38 | 1.12, 0.41 (NS) | 10.34, 10.83 | 1.41, 4.2E-03 |
| *Glud1* | glutamate dehydrogenase 1 | 7.98, 7.42 | -1.46, 3.4E-05 | 8.17, 7.64 | -1.44, 3.1E-05 |
| *Gclc* | glutamate-cysteine ligase, catalytic subunit | 5.15, 8.14 | 9.13, 2.0E-08 | 5.09, 6.85 | 3.63, 2.8E-08 |
| *Gss* | glutathione synthetase | 4.09, 3.87 | -1.11, 0.59 (NS) | 4.13, 3.64 | -1.39, 0.02 |
| *Gpx8* | glutathione peroxidase 8 | 3.58, 2.69 | -1.79, 1.8E-03 | 3.76, 3.27 | -1.37, 0.02 |
| Other Gpx's detected | | | | | |
| *Gpx1* | glutathione peroxidase 1 | 8.57, 8.28 | -1.20, 0.12 (NS) | 8.70, 8.82 | 1.10, 0.50 (NS) |
| *Gpx3* | glutathione peroxidase 3 | -0.97, -0.80 | 1.11, 0.81 (NS) | -1.38, -2.08 | -1.03, 0.97 (NS) |
| *Gpx4* | glutathione peroxidase 4 | 6.95, 6.79 | -1.11. 0.30 (NS) | 7.20, 7.10 | -1.06, 0.66 (NS) |
| *Gpx7* | glutathione peroxidase 7 | -3.87, -1.78 | 2.06, 0.22 (NS) | ND | ND |
| *Gsr* | glutathione-disulfide reductase | 5.82, 6.04 | 1.20, 0.37 (NS) | 5.79, 5.71 | -1.06, 0.59 (NS) |
| Gst's: 17 glutathione-S-transferases were detected in the STR, 16 in the PFC. Those with FDR<0.05 are shown below. | | | | | |
| *LOC501110* | similar to glutathione S-transferase A1 | 1.58, -0.52 | -3.50, 6.2E-05 | ND | ND |
| *Gstm1* | glutathione S-transferase mu 1 | ND | ND | 3.68, 4.31 | 1.56, 0.01 |

| | | | | | |
|---|---|---|---|---|---|
| *Gstm4* | glutathione S-transferase mu 4 | ND | ND | 4.41, 3.85 | -1.47, 1.5E-03 |
| *Gstt1* | glutathione S-transferase theta 1 | 0.58, -0.90 | -2.76, 2.4E-04 | ND | ND |
| *Gstz1* | glutathione S-transferase zeta 1 | 3.57, 2.89 | -1.51, 0.03 | 3.76, 3.20 | -1.45, 0.03 |
| *Mgst3* | microsomal glutathione S-transferase 3 | 3.03, 1.91 | -2.13, 5.6E-05 | 3.10, 2.28 | -1.75, 1.9E-04 |
| *Sod1* | superoxide dismutase 1, soluble | 5.06, 7.00 | 3.98, 2.2E-11 | 5.12, 7.13 | 4.09, 1.1E-09 |
| *Cat* | catalase | 6.42, 5.96 | -1.35, 5.8E-03 | 6.52, 6.34 | -1.13, 0.37 (NS) |

Table S5: Gene expression data for GABA transporters in microglia from the STR at 2 hr and 3 days after METH. NS = Not significant.

| Symbol | Gene Name | 2 hr STR average log2cpm Saline (n=6), METH (n=8) | 2 hr STR Fold Change, FDR | 3 day STR average log2cpm Saline (n=4), METH (n=5) | 3 day STR Fold Change, FDR |
|---|---|---|---|---|---|
| *Slc6a12* | solute carrier family 6 member 12 | -0.32, 3.84 | 16.60, 5.4E-07 | -0.33, 1.07 | 2.76, 3.2E-04 |
| *Slc6a1* | solute carrier family 6 member 1 | 1.27, 1.47 | 1.06, 0.91 (NS) | -0.73, -2.62 | -2.00, 0.54 (NS) |

Table S6: Gene expression data for *Csf1, Csfr1, Il34* in microglia from the STR at 2 hr and 3 days after METH. NS = Not significant.

| Symbol | Gene Name | 2 hr STR average log2cpm Saline (n=6), METH (n=8) | 2 hr STR Fold Change, FDR | 3 day STR average log2cpm Saline (n=4), METH (n=5) | 3 day STR Fold Change, FDR |
|---|---|---|---|---|---|
| *Csf1* | colony stimulating factor 1 | 1.54, 4.91 | 12.52, 7.6E-09 | -0.60, 0.52 | 2.42, 4.6E-03 |
| *Csf1r* | colony stimulating factor 1 receptor | 13.48, 13.62 | 1.10, 0.54 (NS) | 11.73, 11.60 | -1.06, 0.90 (NS) |
| *Il34* | interleukin 34 | -1.52, -1.56 | 1.18, 0.76 (NS) | -2.01, -0.27 | 3.23, 0.02 |
